# Devices in Heart Failure Patients—Who Benefits From ICD and CRT?

**DOI:** 10.3389/fcvm.2019.00111

**Published:** 2019-08-13

**Authors:** Alexander Breitenstein, Jan Steffel

**Affiliations:** Electrophysiology, Department of Cardiology, University Hospital Zurich, Zurich, Switzerland

**Keywords:** heart failure, cardiac resynchronization, implantable cardioverter defibrillator, subcutaneous ICD (S-ICD), imaging

## Abstract

Despite advances in heart failure treatment, this condition remains a relevant medical issue and is associated with a high morbidity and mortality. The cause of death in patients suffering from heart failure is not only a result of hemodynamic failure, but can also be due to ventricular arrhythmias. Implantable cardioverter defibrillators (ICDs) are these days the only tool to significantly reduce arrhythmic sudden death; but not all patients benefit to the same extend. In addition, cardiac resynchronization therapy (CRT) is another tool which is used in patients suffering from heart fialure. Even though both devices have been investigated in large randomized trials, both ICD and CRT remain underutilized in many countries. This brief review focuses on various aspects in this regard including a short overview on upcoming device novelties in the near future.

## Introduction

According to current ESC guidelines, heart failure can be separated into heart failure with preserved ejection fraction (LVEF > 50%; HFpEF), mid-range EF (LVEF 40–49%; HFmrEF) and reduced ejection fraction (LVEF < 40%; HFrHF) (1). The cause of death in patients suffering from heart failure is not only a result of hemodynamic failure but also suddenly and unexpectedly occurring from ventricular electrical disturbances such as ventricular tachycardia and -fibrillation. Implantable cardioverter defibrillators (ICDs) represent the only available tool in our treatment armamentarium proven to prevent arrhythmic sudden cardiac death. In addition, cardiac resynchronization therapy (CRT) is an established treatment option significantly improving both quality of life as well as mortality.

In spite of these proven benefits, both ICD as well as CRT therapy remain underutilized in many countries (2). The reasons are manifold. This brief review focusses on some of the most important aspects in this regard, including continuing medical education regarding the pathophysiology, epidemiology, clinical trial results as well as novel technologies, which are paramount to allow for optimal dissemination of these important therapies and to reduce morbidity and mortality in this fragile patient population.

## Implantable Cardioverter Defibrillator (ICD) Therapy in Patients Suffering From Chronic Heart Failure (CHF)—Of Primary and Secondary Prevention, in Ischemic and Non-ischemic Cardiomyopathy

Patients with heart failure and reduced ejection fraction (LVEF < 35%) are at an increased risk of sudden cardiac death due to ventricular arrhythmias. This risk is highest in those who already suffered from previous ventricular arrhythmic events (3). In this “secondary prevention” situation where no reversible cause such as an acute myocardial infarction can be identified, an ICD is recommended with a class IA indication (if survival > 1 year with good functional status is predicted) according to ESC guidelines (1) (Table 1).

**Table 1:** Current ICD and CRT indications according to the ESC guidelines.

	**Indication ICD**	**Class**
Secondary prevention	In patients with documented VF or haemodynamically not tolerated VT Absence of reversible causes or within 48h after myocardial infarction Reasonable expectation of survival with a good functional status > 1 year	IA
Primary prevention	Severely impaired LV function (≤ 35 %) With NYHA II-IVa	
	Despite optimal medical treatment for ≥ 3 months	
	Ischemic cardiomyopathy Non-ischemic cardiomyopathy	IA IB
	**Indication CRT**	**Class**
	Severely impaired LV function (≤ 35 %) NYHA II-IVa Despite optimal medical treatment for ≥ 3 months	
	With LBBB and QRS duration > 150 ms With LBBB and QRS duration 130-149 ms	IA IB
	With non-LBBB and QRS duration > 150 ms With non-LBBB and QRS duration 130-149 ms	IIA IIB

The situation is more complex in primary prevention. Even though current guidelines recommend the implantation of an ICD in patients suffering from heart failure with an LVEF ≤ 35% despite at least 3 months of optimal medical treatment, a NYHA class II-IVa and a predicted survival of > 1 year, there seems to be a discrepancy of the beneficial effect of an ICD depending on the underlying heart disease. Indeed, patients with ischemic heart disease have a class IA indication, while in the non-ischemic population the indication level is IB (Table 1). Two large trials investigated the role of an ICD in the primary prevention context: The Sudden Cardiac Death in Heart Failure Trial (SCD-HeFT) (4) and the Multicenter Automatic Defibrillator Implantation Trial II (MADIT-II) (5). Both trials demonstrated a mortality benefit in patients with severely reduced LVEF (≤ 30% in the MADIT-II trial, ≤ 35% in SCD-HeFT). In MADIT-II, which enrolled patients with ischemic heart disease and a previous myocardial infarction, the ICD offered a relative mortality reduction of 31% over 5 years. SCD-HeFT on the other hand recruited a mixed population of ischemic and non-ischemic cardiomyopathy. Subgroup analyses did not show a difference in the outcome of total mortality reduction between ischemic and non-ischemic heart disease indicating a similar effect in both patient population. In contrast, the more recent randomized Danish Study to Assess the Efficacy of ICDs in Patients with Non-ischemic Systolic Heart Failure on Mortality (DANISH) trial investigated the role of primary prevention ICDs exclusively in non-ischemic cardiomyopathy; it demonstrated a significant reduction in sudden cardiac death in patients with non-ischemic heart disease with an LVEF ≤ 35%—but without an effect on all-cause mortality, the predefined primary endpoint (6).

**Figure 1:**
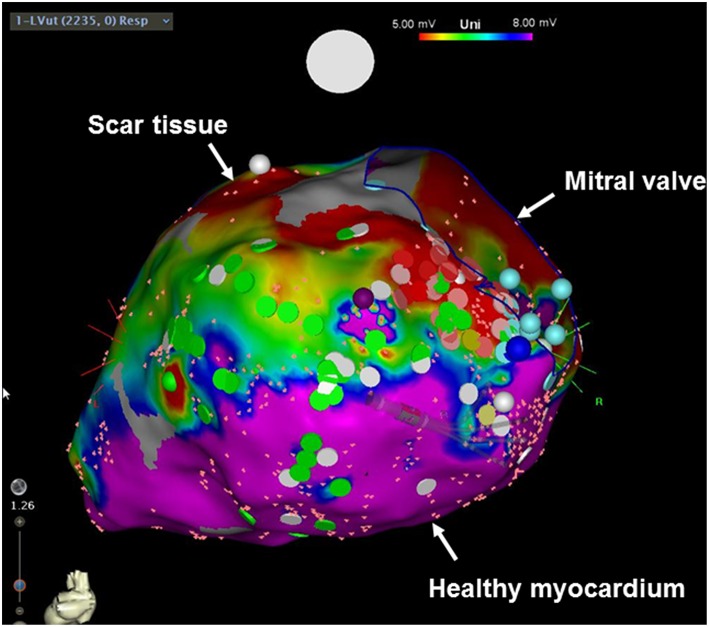
Electroanatomical voltage mapping of the left ventricle (posterior view). Red areas indicate scar tissue, whereas pink areas demonstrate healthy myocardium.

How do these results fit together? Do we need to rethink the indication for primary prevention ICD implantation in the entire non-ischemic population? Even though a recent survey of the European Heart Rhythm Association (EHRA) has shown that already 4 months after the publication of the DANISH trial, nearly 50% of physicians changed their current practice in recommending an ICD implantation in a non-ischemic cardiomyopathy patient (7), a meta-analysis has shown a benefit of primary prevention ICDs even regarding total mortality (8). What to do in light of these controversial data? Rather than jumping to conclusions and withholding an ICD on a general basis in this population, it may be worthwhile to take a look at the data in a little bit more detail. Indeed, improving patient selection in non-ischemic heart disease may be the way to go to maximize the benefit of ICD therapy. Subgroup analyses of the DANISH trial have shown that younger patients with an age of < 70 years or those with less severe heart failure (as indicated by lower NT-proBNP levels) indeed demonstrated a reduction in all-cause mortality (6, 9). These data imply that using just the LVEF alone to predict whether an individual patient will more likely die from pump failure vs. ventricular arrhythmias (10–12) may be insufficient. On a more general level, these results further indicate that our current way of risk-stratification is insufficient and that a more refined way of looking at things is warranted. The Seattle Heart Failure Model, for example, incorporates various risk factors including age as well as laboratory parameters to predict mortality in heart failure patients, and may represent an attractive model for patient selection for an ICD. Another promising strategy is to assess the potential proarrhythmogenic myocardial substrate. Late gadolinium enhancement (LGE) on cardiac MRI corresponds to areas of myocardial fibrosis (13), and reports have shown that patients with LGE exert a higher risk for sudden cardiac death and ventricular arrhythmias (14). Especially mid-wall LGE was associated with a substantial nine-fold increase in the rate of sudden cardiac death/aborted sudden cardiac death in patients with dilated cardiomyopathy and a LVEF ≥ 40% (15). In addition to CMRI, electroanatomical mapping (EAM) is a currently widespread way of invasively investigating myocardial scaring (Figure 1). By measuring local electrical properties in the context of an electrophysiological study, areas of scar tissue can be differentiated from healthy myocardium (16). Even though both imaging modalities display great promise, their value needs to be prospectively tested in randomized trials. In addition to imaging modalities, various other markers of increased arrhythmia risk can potentially be incorporated in the ICD decision making process such as genetic testing, circulating biomarkers, non-invasive electrophysiological testing etc.

**Figure 2:**
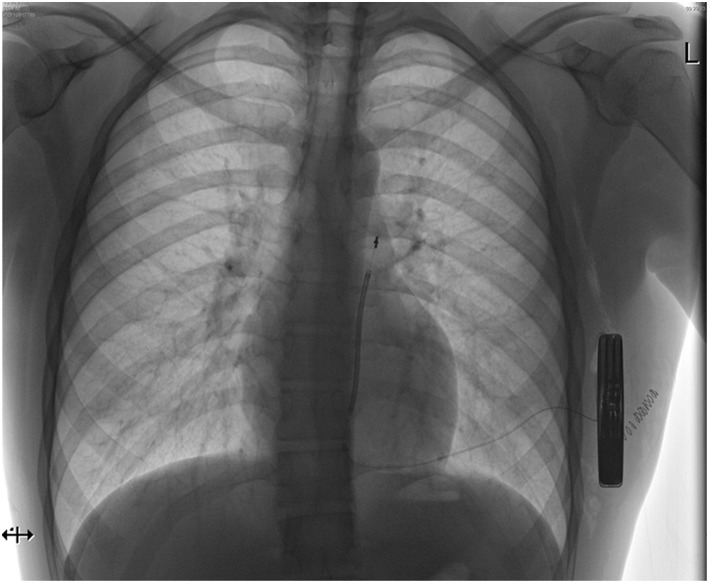
Antero-posterior chest X-ray after subcutaneous ICD (S-ICD) implantation.

On the other hand, it needs to be kept in mind that the reduction in sudden cardiac death from an ICD in this population not translating into a total-mortality benefit may not only be because of an excess in non-arrhythmogenic deaths—it may also be due to complications and morbidity of the device itself. Therefore, further investigations and developments in improving device technology are essential. The subcutaneous implantable cardioverter defibrillator (S-ICD) represents a novel model of an ICD designed to reduce the occurrence as well as the associated morbidity (and mortality) of one of the most relevant lifetime risks of transvenous device systems: infection. The generator in a S-ICD is located at the left lateral chest in the space between the anterior serratus and the latissimus dorsi muscles. The lead is tunneled within the subcutaneous tissue toward the xiphoid process and from advanced alongside the sternum cranially (Figure 2). Large randomized trials are on its way, but registries are very promising with lower lead complications and a higher lead survival over time in the SICD population (17). As such, recent ESC guidelines recommend a subcutaneous ICD with a class IIa indication as an alternative to transvenous ICDs in the absence of contraindication (18). A subcutaneous defibrillator is not suitable if patients are in need for bradycardia pacing or cardiac resynchronization as well as overdrive pacing; however, the solution of most these problems, however, appears to be only a matter of time, since a combination of the S-ICD with a leadless pacemaker will enter into clinical trials in the near future. Furthermore, patients need to undergo a pre-procedure sensing vector screening to ensure adequate sensing of the QRS complex and T wave to avoid under- as well as T wave oversensing; at least one out of 3 sensing vectors needs to have appropriate sensing in a supine and sitting or standing posture.

## Cardiac Resynchronisation Therapy (CRT)—Of “Responders” and “Non-Responders”

Cardiac resynchronization therapy has fundamentally changed the treatment of patients suffering from heart failure. Impaired left ventricular function often results in ventricular conduction disease, which secondarily leads to electrical and mechanical ventricular dyssynchrony further impairing hemodynamics of the ventricle (19). Disrupting this vicious is the goal of biventricular pacing in CRT.

According to current guidelines, a CRT device is indicated in patients with stable, symptomatic (NYHA II-IVa) systolic heart failure despite 3 months of optimal medical treatment, an LVEF ≤ 35% and a QRS duration of ≥ 130 ms (1). This indications results from large randomized trials investigating the role of cardiac resynchronization in patients with severely symptomatic heart failure (mostly NYHA III) and a QRS duration of ≥ 120 ms: The Comparison of Medical Therapy, Pacing, and Defibrillation in Heart Failure (COMPANION) (20) and the Cardiac Resynchronisation-Heart Failure (CARE-HF) (21) trial. Both studies have demonstrated that biventricular stimulation in this population reduced total mortality up to 36%, which was later confirmed by registry data and meta-analyses (22–24). Subsequently, the benefit of CRT has been extended to patients with mild heart failure symptoms (NYHA II). In the Multicenter Automatic Defibrillator Implantation Trial with Cardiac Resynchronization Therapy (MADIT-CRT), heart failure patients with LVEF ≤ 30% and NYHA I-II as well as a QRS duration of > 130 ms were enrolled (25). Total mortality in the CRT group was 34% lower as compared to only medically treated patients. This finding is support by data from the Resynchronization-Defibrillation for Ambulatory Heart Failure (RAFT) trial, which has shown in patients with LVEF of ≤ 30%, a QRS duration of > 120 ms and NYHA II-III that CRT reduces total mortality by 25% (26).

**Figure 3:**
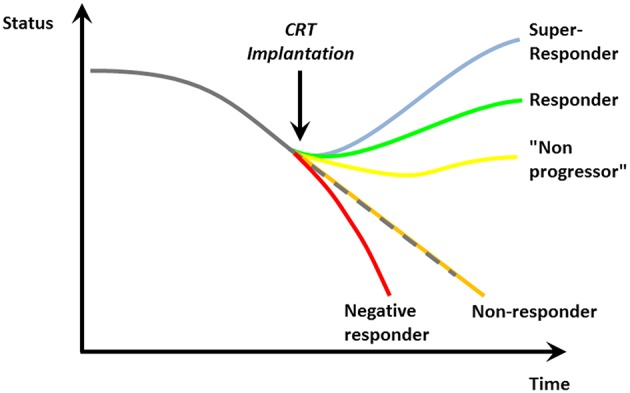
Possible responses to cardiac resynchronization [Reproduced with permission from Circulation (36)].

In spite of these impressive results, a couple of aspects in the context of CRT need to be discussed:

1. From the MADIT-CRT and RAFT trials, there is insufficient data to recommend a CRT in patients with NYHA class I (25, 26).
2. Even though a CRT device reduces mortality in correctly selected patients, it has no benefit and may cause harm if implanted in the wrong patients. The EchoCRT trial demonstrated no benefit and a signal for increased mortality if a CRT device is implanted in patients with a narrow QRS complex (despite mechanical ventricular dyssynchrony as assessed by echocardiography) (27).
3. Guidelines judge a CRT device implantation in patients with a LBBB with class I indication (IA if QRS width if > 150 ms, IB if QRS duration is 130–149 ms), whereas non-LBBB situations get a class II indication. Patients with LBBB indeed seem to have a more severe type of LV electrical dyssynchrony as compared to non-LBBB situation (28, 29) and are likely to benefit more from cardiac resynchronization. As such, in the MADIT-CRT trial, a pre-defined subgroup analysis demonstrated that only patients with LBBB derived a greater benefit from CRT regarding heart failure event-free survival (25, 26, 30). This finding is supported by a meta-analysis of the above mentioned trials showing no benefit of CRT in non-LBBB patients (24). One needs to keep in mind that none of the pivotal trials investigating the role of CRT in heart failure patients used QRS morphology as an inclusion criterion (in contrast to QRS duration).
4. Atrial fibrillation (AF) was an exclusion criterion in the vast majority of CRT trials (except for the RAFT study), even though AF and heart failure regularly co-exists and patients with AF together with heart failure exhibit a worse prognosis (31, 32). Due to fast, irregular atrioventricular conduction, AF not rarely limits the benefit of a CRT device compared to sinus rhythm (33). However, if atrial fibrillation conduction is interrupted by atrioventricular node ablation, the benefit of a CRT device—at least in non-randomized trials and meta-analysis—was shown to be similar to patients without AF (34, 35).
5. On the flip-side one needs to be aware that up to one-third of patients with a CRT device does not show a benefit over time (so-called “non-responders”; Figure 3) (37). However, there is no standard consensus on the definition of “response” and “non-response”: While some use clinical parameters (mortality, heart failure hospitalization, NYHA class) (19, 38), others use echocardiographic measurements (increase in LVEF, decrease in endsystolic volume) (39) or a combination. Not rarely, there is a discrepancy between clinical and echocardiographic responses (40). A typical example is the response of CRT in patients with ischemic heart disease. Patients with prior myocardial infarction are known to demonstrate less of an echocardiographic response/remodeling to CRT (41, 42). However, the benefit of CRT in the pivotal trials (CARE-HF and COMPANION) did not depend on the underlying heart disease (20, 21). On the contrary, since patients suffering from ischemic heart disease have a worse prognosis with a higher absolute rate of events, their absolute risk reduction may be even higher (43)—and, as a result, the “number needed to treat” even lower.

Finally, the “response” to CRT is a very relative measure. Apart from non-responders, as mentioned above, patients may stabilize their cardiac function and clinical course. We previously coined these individuals “non-progressors” (36). On a more fundamental level, the lack of visible improvement in LVEF or clinical status after CRT implantation may lead physicians to distrust CRT as a valuable way to reduce morbidity and mortality—leading to the observed lower-than-expected implementation rates (2). We tend to forget in these cases that CHF is a chronically progressive, malignant disease and stabilization of its course—sometimes over years—often incorrectly termed as “non-response” indeed indicates a significant benefit of CRT for this vulnerable patient population.

On the other side of the spectrum, some patients exhibit a huge benefit of CRT and may even normalize their LV function (so-called “super-responders”; Figure 3) (36). Every effort has to be undertaken to achieve maximum benefit from a CRT device. Even though echocardiographic assessment of ventricular dyssynchrony is not a reliable parameter to predict outcome to CRT (27, 44), pre-implantation imaging may become more important to identify optimal position for the LV lead (45–47). During the implantation, it is crucial to place the lead—if possible—at the localization with the longest electrical delay to potentially offer a higher level of resynchronization (48). Optimal device programming by experts in device follow-up and troubleshooting post-implantation and during the further course is furthermore essential to provide maximum benefit (49). Even though routinely optimizing device intervals after CRT implantation has not been shown to be of clinical benefit (50), it may be relevant in “non-responders,” negative responders and those experiencing clinical events and complications. In addition, device-based algorithms to optimize CRT response such as AdaptivCRT (51, 52) or SyncAV (53) may become more important in the near future.

In addition to biventricular pacing, other pacing techniques are under investigation which potentially may further improve the outcome in patients with heart failure. One such strategy is His-bundle pacing (HBP); indeed, HBP in the context of bradycardia indications has been shown to be a safe and successful option for patients with high burden of chronic ventricular pacing (54). The role of HBP in heart failure patients is currently investigated in various trials (HOPE-HF trial, His-SYNC trial) (55). In how far this will become a standard also outside expert centers with specific expertise remains to be proven in large outcome studies.

## Conclusion

Implantable devices such as ICDs and CRTs are a cornerstone in the modern treatment of heart failure patients, reducing morbidity and mortality in this population. Continuing medical education regarding the pathophysiology, epidemiology, clinical trial results and novel technologies are of paramount importance to optimize patient selection and individual benefit. Ongoing and future studies and technological advances are aiming to improve patients' prognosis even further. Their implementation, however, will be the task of all stakeholders involved including cardiologists, general practitioners and—increasingly importantly—the patient him/herself. Only by a collaborative effort will we be able to move the field forward, and to further develop concepts to improve the prognosis of patients with heart failure.

## Author Contributions

All authors listed have made a substantial, direct and intellectual contribution to the work, and approved it for publication.

### Conflict of Interest Statement

AB has received consultant and/or speaker fees from Abbott, Bayer, Biosense Webster, Biotronik, Boston Scientific, Bristol-Myers Squibb, Daiichi Sankyo, Medtronic, Novartis, and Spectranetics/Philipps. JS has received consultant and/or speaker fees from Abbott, Amgen, Astra-Zeneca, Atricure, Bayer, Biosense Webster, Biotronik, Boehringer-Ingelheim, Boston Scientific, Bristol-Myers Squibb, Daiichi Sankyo, Medscape, Medtronic, Merck/MSD, Novartis, Pfizer, Sanofi-Aventis, WebMD, and Zoll. He reports ownership of CorXL. JS has received grant support through his institution from Abbott, Bayer Healthcare, Biosense Webster, Biotronik, Boston Scientific, Daiichi Sankyo, and Medtronic.
